# Neuroactive Steroids and Cognitive Functions in First-Episode Psychosis Patients and Their Healthy Siblings

**DOI:** 10.3389/fpsyt.2019.00390

**Published:** 2019-06-18

**Authors:** Pavel Knytl, Veronika Voráčková, Aneta Dorazilová, Mabel Rodriguez, Aneta Cvrčková, Edita Kofroňová, Martin Kuchař, Zuzana Kratochvílová, Petra Šustová, Silvie Čerešňáková, Pavel Mohr

**Affiliations:** ^1^National Institute of Mental Health, Klecany, Czechia; ^2^Third Faculty of Medicine, Charles University, Prague, Czechia; ^3^Faculty of Arts, Masaryk University, Brno, Czechia; ^4^Faculty of Arts, Charles University, Prague, Czechia; ^5^Faculty of Social Studies, Masaryk University, Brno, Czechia; ^6^University of Chemistry and Technology, Prague, Czechia; ^7^First Faculty of Medicine, Charles University, Prague, Czechia

**Keywords:** neuroactive steroids, cognition, endophenotype, siblings, psychosis

## Abstract

**Background:** Neuroactive steroids (NAS) affect neurotransmitter systems and cognition; thus, they play role in etiopathogenesis of psychiatric disorders.

**Aims:** The primary aim was to examine cognition and effects of NAS on cognitive functioning in first-episode psychosis patients and in their healthy siblings. The secondary aims were to verify whether cognitive deficit is an endophenotype of psychosis and whether higher NAS levels represent a high-risk factor for psychosis.

**Methods:** Studied participants were 1) patients with first episode of psychosis, 2) healthy siblings of the patients, and 3) matching healthy controls. Study procedures included administration of a battery of neuropsychological tests assessing six cognitive domains and examination of NAS plasma levels [cortisol (CORT), 11-deoxycorticosterone (DOC), testosterone (TEST), dehydroepiandrostendione (DHEA), dihydrotestosterone (DHT), and progesterone (PROG)].

**Results:** A total of 67 subjects were analyzed (16 patients, 22 siblings, and 29 controls). Significant group differences were found in most of the cognitive domains; the patients had the lowest scores. The Kruskal–Wallis test revealed significant group differences in CORT levels (*p* < 0.01), TEST (*p* < 0.01), and DHT (*p* < 0.001); no difference was found in PROG, DHEA, and DOC. All cognitive domains, except for attention, were affected by the NAS levels. CORT levels of patients correlated with speed of processing (*r* = 0.55) and working memory (*r* = 0.52), while PROG levels correlated with abstraction (*r* = −0.63). In siblings, there was a negative correlation between TEST levels and verbal memory (*r* = −0.51) and PROG with attention (*r* = −0.47).

**Conclusions:** Our results verified that individual domains of cognitive deficit (abstraction and verbal memory) can be considered as an endophenotype of psychosis. Higher levels of cortisol and testosterone in siblings are consistent with high-risk states for psychosis. Multiple interactions between NAS and cognitive functioning, particularly memory functions, were observed. Study limitations (small sample size and administration of antipsychotic medication) did not allow us to establish unequivocally NAS as an endophenotype.

## Introduction

Complex etiopathological mechanisms of schizophrenia are still poorly understood and their key features are yet to be identified. The widely cited dopaminergic theory (1) seems to be just an ending point of several pathophysiological processes in which other neurotransmitter systems (e.g., glutamatergic, GABAergic, and serotoninergic) are involved, as well (2–4).

In addition to other factors, neurotransmission is significantly affected by steroid substances synthetized in the central nervous system (CNS), labeled as “neurosteroids” (5, 6). A more general term, “neuroactive steroids” (NAS) is used for all substances that interact with the CNS in two different modes. First, genomically, through binding with intracellular steroid receptors, NAS can change gene expression. Second, interacting with membrane neurotransmitter receptors, NAS can modulate psychopathology of psychiatric disorders (7, 8). NAS may exhibit therapeutic potential, and their antidepressant, anxiolytic, and antipsychotic effects have been reported (9–11). Targeting NAS metabolism is considered as a novel therapeutic approach (12–14). Alterations of NAS are currently being studied intensively to investigate their role in the pathophysiology of schizophrenia, with an accumulating amount of evidence suggesting their involvement (12, 15, 16). However, the findings are not consistent, mostly due to the treatment, age, or gender effects (17–19).

Studies showed that early intervention can improve outcome of schizophrenia patients (20, 21). Therefore, prodromal phases of psychotic disorders are the focus of current research efforts (22). Healthy siblings of schizophrenia patients are a well-defined population at higher risk of developing illness (23). One promising approach on how to investigate etiopathogenesis of psychiatric disorders is through endophenotypes (24). Endophenotypes are state independent, observed in actively manifested illness or in remission and are present in healthy relatives of patients, but not in the general population.

Various candidate endophenotypes of schizophrenic disorder have been proposed, e.g., structural changes of the white and grey brain matter, biochemical blood markers, neurophysiological measures, or impairment of cognitive and social functioning (25). Cognitive deficit was also consistently observed in a population at risk of psychosis (26). A recent meta-analysis found a correlation between cognitive impairment and metabolic syndrome, suggesting an effect of metabolic alteration on cognition (27).

Several studies linked NAS to cognitive functioning of schizophrenia spectrum patients (28). NAS were also examined as candidate adjunctive agents for treatment of cognitive impairment in schizophrenia (29, 30). However, there are so far no data on the relationship of NAS and cognition among high-risk individuals.

The primary aim of our study was to examine the differences in cognitive performance of first-episode patients with psychosis, their siblings, and healthy controls. The secondary goal was to examine the effect of NAS on cognition, in order to verify whether cognitive deficit is an endophenotype of psychosis and whether higher levels of NAS represent a high-risk factor for psychotic disorder.

## Materials and Methods

### Study Sample

The study was conducted at the National Institute of Mental Health, Czechia. The study sample consisted of three groups of subjects: (1) first-episode psychosis patients; (2) their unaffected siblings; and (3) matching healthy controls. Patients met the *International Statistical Classification of Diseases and Related Health Problems*, 10th revision (*ICD-10*), criteria for schizophrenia (F20.x), acute psychosis (F23.x), or schizoaffective disorder (F25.x), first episode (31). Siblings and the control group were assessed with the Mini International Neuropsychiatric Interview (MINI) to rule out any current or past psychiatric disorders. Exclusion criteria in all groups were organic brain disorder, neurological or endocrine disease, substance dependence, intellectual disability, motor or perceptual handicap, and incapacity to provide informed consent. The study protocol was approved by the local ethical committee, and all subjects signed an informed consent form.

### Study Procedures

Basic demographic and clinical data, sex, age, education, medical history, family history, and duration of untreated psychosis were recorded. Severity of psychotic symptoms in the patient group was assessed with the Positive and Negative Syndrome Scale (PANSS) and the Clinical Global Impression (CGI). Patients were assessed during the non-acute phase of their illness (CGI ≤ 4). The MINI was used to examine psychiatric comorbidity among patients and to verify the absence of psychiatric disorder in siblings and healthy controls.

Cognitive tests were administered by trained psychologists, using both pencil-paper and computer methods. Composition of the neuropsychological battery was based on the expert consensus and our previous research (32, 33). The battery evaluated cognitive functions in the following six domains: psychomotor speed/speed of processing [tested with Verbal Fluency Test; Stroop Test: word, color; Trail Making Test (TMT) A; Wechsler Adult Intelligence Scale (WAIS-III): digit–symbol coding]; attention (Continuous Performance Task); working memory/flexibility [WAIS-III: digit span; WAIS-III: letter–number sequencing; Wechsler Memory Scale (WMS-III): spatial span; TMT-B; Stroop Test: color word]; verbal memory (Auditory Verbal Learning Test, WMS-III: logical memory); visual memory (Rey–Osterrieth Complex Figure Test), and abstraction (WAIS-III: similarities; WAIS-III: comprehension; Picture arrangement; Wisconsin Card Sorting Test; Tower of London). For a detailed description and calculation of the composition and consistency of cognitive domains, see Rodriguez et al. ( 33). Participants were tested in two consecutive sessions with a break in between, and maximum time of psychological assessment was 120 min.

All subjects had their blood samples taken after 12 h of fasting; females were in the follicular phase of their menstrual cycle. NAS that previously showed an impact on the cognition of schizophrenia patients were assessed (28, 34–38). Plasma levels of cortisol (CORT), 11-deoxycorticosterone (DOC), testosterone (TEST), dehydroepiandrostendione (DHEA), dihydrotestosterone (DHT), and progesterone (PROG) were analyzed with liquid chromatography and mass spectroscopy in laboratories of the University of Chemistry and Technology in Prague.

### Statistical Analysis

Fisher’s exact test was used to detect possible differences in distribution of sex and education between the groups. Non-parametric Kruskal–Wallis test and epsilon squared as effect size were used to assess the age differences to compare cognitive performance between the groups and for the group comparison of the NAS levels. The Dunn test was used for the *post hoc* multiple comparison; *p*-values were adjusted with the Benjamini–Hochberg method. Non-parametric version of analysis of covariance (ANCOVA) was used to examine possible differences in the NAS levels between the groups in relation to cognitive performance. For statistical analyses, R software version 3.3.2 was used.

## Results

### Study Sample

The total number of enrolled subjects was 74 (22 patients, 23 siblings, and 29 controls). Six patients and one sibling were excluded from the analyses due to the following reasons: manifested psychotic symptoms during cognitive testing, non-native Czech speaker, history of multiple episodes, and incorrect diagnosis. Analyzed study sample included 67 subjects: 16 first-episode patients, 22 siblings, and 29 healthy controls. There was no significant group difference in sex ratio (Fisher’s exact test: *p* = 0.535; Cramer’s *V* = 0.14), education (Fisher’s exact test: *p* = 0.09; Cramer’s *V* = 0.32), or age [Kruskal–Wallis test: H(2) = 2.95, *p* = 0.23; ε^2^ = 0.05]. For details, see **Table 1**.

**Table 1:** Study sample, demographic characteristics.

		Patients (*n* = 16)	Siblings (*n* = 22)	Controls (*n* = 29)	All (*n* = 67)
**Sex **	Male (%)	8 (50)	15 (68)	17 (59)	40 (60)
	Female (%)	8 (50)	7 (32)	12 (41)	27 (40)
**Age (years)**	Mean (SD)	25.81 (5.56)	29.27 (7.32)	29.38 (6.48)	28.49 (6.65)
	Median	26.5	29	29	28
**Education **	Elementary (%)	3 (19)	0 (0)	1 (3)	4 (6)
	Secondary, without graduation (%)	3 (19)	6 (27)	2 (7)	11 (16)
	Secondary, graduated (%)	4 (25)	11 (50)	15 (52)	30 (45)
	University (%)	6 (37)	5 (23)	9 (31)	20 (30)
	College (%)	0 (0)	0 (0)	2 (7)	2 (3)

Data on the patients’ psychiatric history (duration of illness, duration of untreated psychosis, length of antipsychotic treatment, and drug dosage in chlorpromazine equivalents) are shown in **Table 2**. Except for one patient, all of them were treated with antipsychotic medication. Fourteen were given oral monotherapy (for 7 subjects olanzapine 10–30 mg p.d.; 4 risperidone 3–4.5 mg p.d.; 1 aripiprazole 15 mg p.d.; 1 clozapine 175 mg p.d.; and 1 patient quetiapine 500 mg p.d). Four patients were prescribed a combination of two oral drugs (olanzapine 25 mg + haloperidol 6 mg; olanzapine 30 mg + aripiprazole 30 mg; and aripiprazole 15 mg + clozapine 150 mg), and four subjects were administered long-acting injectable antipsychotics (for 2 patients paliperidone 150 mg monthly; 1 olanzapine 300 mg biweekly; and 1 flupenthixol 40 mg monthly).

**Table 2:** Psychiatric history of the patients.

	Mean (SD)	Median
**Duration of illness (months)**	8.70 (8.81)	5
**Duration of untreated psychosis (months)**	3.17 (4.45)	1
**Length of antipsychotic treatment (months)**	5.21 (8.68)	1.38
**Antipsychotic dosage (chlorpromazine equivalents in mg/day)**	330 (220.30)	297

### Cognition

Mean and median values of *Z*-scores for each of the cognitive domains are summarized in **Table 3**. Significant group differences were found in most of the cognitive domains, with the exception of attention. Kruskal–Wallis test: visual memory [H(2) = 12.67, *p* < 0.01, ε^2^ = 0.19]; verbal memory [H(2) = 15.38, *p* < 0.001, ε^2^ = 0.24]; abstraction [H(2) = 10.09, *p* < 0.01, ε^2^ = 0.15]; psychomotor speed [H(2) = 6.66, *p* < 0.05, ε^2^ = 0.1]; working memory [H(2) = 8.75, *p* < 0.05, ε^2^ = 0.13]; attention [H(2) = 1.39, *p* > 0.05, ε^2^ = 0.02].

**Table 3:** Descriptive characteristics of cognitive domains (z-scores).

		Mean (SD)	Median
**Visual memory^a^**	Patients	−0.74 (0.55)	−0.84
	Siblings	0.13 (0.8)	0.34
	Controls	0 (0.73)	0.02
**Verbal memory^b^**	Patients	−1.34 (1.33)	−0.85
	Siblings	−0.67 (0.84)	−0.8
	Controls	0 (0.79)	−0.01
**Abstraction^a^**	Patients	−1.17 (1.58)	−0.46
	Siblings	−0.7 (0.9)	−0.78
	Controls	0 (0.74)	0.03
**Psychomotor speed/speed of processing^c^**	Patients	−0.7 (0.99)	−0.53
	Siblings	−0.14 (0.92)	0
	Controls	0 (0.57)	0.13
**Attention**	Patients	−0.22 (0.8)	0.09
	Siblings	0.08 (0.62)	0.25
	Controls	0 (0.74)	0.16
**Working memory/flexibility** **^c^**	Patients	−0.96 (1.48)	−0.6
	Siblings	−0.42 (0.94)	−0.31
	Controls	0 (0.57)	−0.08

Group differences: ^a^p < 0.01; ^b^p < 0.001; ^c^p < 0.05.

Results of the *post hoc* Dunn test with the Benjamini–Hochberg correction (median values listed in **Table 3**) revealed that visual memory performance of controls was statistically superior to that of patients (*p* < 0.05), performance of siblings was significantly better than that of patients (*p* < 0.05), and no difference between siblings and controls was observed. In verbal memory, controls performed significantly better than patients (*p* < 0.001) and siblings (*p* < 0.01), but no difference was detected between patients and siblings. In abstraction, controls performed significantly better than patients (*p* < 0.05) and siblings (*p* < 0.05), but no difference was found between patients and siblings. In psychomotor speed/speed of processing, control subjects outperformed significantly patients (*p* < 0.05), while no significant difference was observed between controls and siblings or between patients and siblings. In working memory/flexibility, control subjects performed significantly better than patients (*p* < 0.05), but no significant differences were detected between controls and siblings and between patients and siblings.

### Neuroactive Steroids

Mean and median values of plasma levels of the analyzed NAS (CORT, DOC, TEST, DHEA, DHT, and PROG) are summarized in **Table 4**. Kruskal–Wallis test revealed significant differences between the groups in CORT levels [H(2) = 11.77, *p* < 0.01, ε^2^ = 0.18], TEST [H(2) = 11.57, *p* < 0.01, ε^2^ = 0.18] and DHT [H(2) = 16.69, *p* < 0.001, ε^2^ = 0.25]. No differences were found in levels of PROG [H(2) = 0.30, *p* > 0.05, ε^2^ = 0.005], DHEA [H(2) = 3.66, *p* > 0.05, ε^2^ = 0.06], and DOC [H(2) = 5.19, *p* > 0.05, ε^2^ = 0.08].

**Table 4:** Plasma levels of the selected neuroactive steroids (ng/ml).

		Mean (SD)	Median
**Cortisol (CORT)^a^**	Patients	174.17 (85.1)	181.17
	Siblings	236.07 (86.22)	245.73
	Controls	153.65 (59.46)	153.74
**Testosterone (TEST)^a^**	Patients	3.78 (4.21)	1.64
	Siblings	8.13 (6.03)	8.61
	Controls	4.45 (3.93)	4.71
**Progesterone (PROG)**	Patients	0.12 (0.1)	0.11
	Siblings	0.14 (0.06)	0.12
	Controls	0.13 (0.1)	0.14
**Dehydroepiandrostendione (DHEA)**	Patients	10.71 (5.28)	11.72
	Siblings	13.53 (6.77)	13.05
	Controls	16.63 (10.09)	14.81
**Dihydrotestosterone (DHT)^b^**	Patients	0.45 (0.28)	0.41
	Siblings	1.07 (0.56)	0.93
	Controls	0.68 (0.31)	0.64
**11-Deoxycorticosterone (DOC)**	Patients	0.08 (0.04)	0.08
	Siblings	0.06 (0.02)	0.05
	Controls	0.05 (0.04)	0.05

Group differences: ^a^p < 0.01; ^b^p < 0.001.

*Post hoc* multiple comparisons (Dunn test) and adjustments with the Benjamini–Hochberg correction (median values listed in **Table 4**) found that CORT levels were significantly higher in siblings than in controls (*p* < 0.01), while no differences in CORT were detected between patients and siblings and between patients and controls. TEST levels were significantly higher in siblings than in both controls (*p* < 0.01) and patients (*p* < 0.01), but no difference was observed between patients and controls. Levels of DHT were significantly higher in siblings than in controls (*p* < 0.05) and in patients (*p* < 0.001), but DHT levels were significantly higher in controls than in patients (*p* < 0.05).

### Effect of Neuroactive Steroids on Cognition

The relationship of NAS with cognition across the study groups was tested with non-parametric ANCOVA. For CORT, equality was rejected for verbal memory (test of equality: *p* = 0.0185), visual memory (*p* = 0.0374), and working memory (*p* = 0.0179) but not for speed of processing (*p* = 0.0744), abstraction (*p* = 0.1991), and attention (*p* = 0.2856). Parallelism was rejected for verbal memory (test of parallelism: *p* = 0.0097) and working memory (*p* = 0.012) but not for visual memory (*p* = 0.1092).

In TEST analysis, equality was rejected for verbal memory (*p* = 0.0303), visual memory (*p* = 0.0109), and abstraction (*p* = 0.0118) but not for speed of processing (*p* = 0.1636), working memory (*p* = 0.0563), and attention (*p* = 0.4698). Parallelism was rejected for verbal memory (*p* = 0.0177), visual memory (*p* = 0.0108), and abstraction (*p* = 0.0095) but not for speed of processing (*p* = 0.1096) and attention (*p* = 0.4976).

In PROG, equality was rejected for visual memory (*p* = 0.027) and abstraction (*p* = 0.0108) but not verbal memory (*p* = 0.1665), speed of processing (*p* = 0.25), working memory (*p* = 0.3633), and attention (*p* = 0.8943). Parallelism was rejected for abstraction (*p* = 0.0076) but not visual memory (*p* = 0.0736).

In DHEA, equality was rejected for verbal memory (*p* = 0.0456) and visual memory (*p* = 0.016) but not for speed of processing (*p* = 0.593), abstraction (*p* = 0.0814), attention (*p* = 0.5217), and working memory (0.6568). Parallelism was rejected for verbal memory (*p* = 0.0445) and visual memory (*p* = 0.024).

In DHT, equality was rejected for verbal memory (*p* = 0.0055), visual memory (*p* = 0.0149), speed of processing (*p* = 0.0146), abstraction (*p* = 0.0063), and working memory (*p* = 0.022) but not for attention (0.5326). Parallelism was rejected for verbal memory (*p* = 0.0054), visual memory (*p* = 0.0143), speed of processing (*p* = 0.0115), abstraction (*p* = 0.0066), and working memory (*p* = 0.0112).

In DOC, equality was rejected for verbal memory (*p* = 0.0095) and visual memory (*p* = 0.024) but not for speed of processing (*p* = 0.1703), abstraction (*p* = 0.1164), attention (*p* = 0.524), and working memory (0.793). Parallelism was rejected for verbal memory (*p* = 0.0081) and visual memory (*p* = 0.044).

Significant positive correlations of NAS with cognition in patients were found between the cortisol levels and speed of processing (*r* = 0.55), and working memory (*r* = 0.52), and negative correlations were found between progesterone and abstraction (*r* = −0.63) (**Table 5**). In siblings, there was a negative correlation between testosterone and verbal memory (*r* = −0.51) and between progesterone and attention (*r* = −0.47) (**Table 6**). No significant correlation was observed in healthy controls.

**Table 5:** Correlations between neurosteroid levels and domains of cognition—patients.

	CORT	TEST	PROG	DHEA	DHT	DOC
**Verbal memory**	−0.003	−0.19	−0.47	0.09	−0.45	0.25
**Visual memory**	0.19	0.22	−0.25	−0.12	0.40	0.25
**Processing speed**	0.55*	−0.08	−0.14	0.02	−0.15	−0.20
**Abstraction **	0.02	0.06	−0.63*	−0.26	−0.25	−0.12
**Attention **	0.32	0.25	−0.17	−0.45	0.05	0.39
**Working memory/flexibility**	0.52*	0.37	0.21	−0.24	−0.20	0.05

CORT, cortisol; TEST, testosterone; PROG, progesterone; DHEA, dehydroepiandrostendione; DHT, dihydrotestosterone; DOC, 11-deoxycorticosterone. *Significant correlation.

**Table 6:** Correlations between neurosteroid levels and domains of cognition—siblings.

	CORT	TEST	PROG	DHEA	DHT	DOC
**Verbal memory**	−0.095	−0.51*	−0.02	−0.19	−0.12	0.15
**Visual memory**	0.021	−0.06	−0.31	−0.35	−0.10	−0.10
**Processing speed**	0.038	−0.24	−0.32	−0.07	−0.30	−0.19
**Abstraction **	0.024	0.01	−0.36	−0.02	−0.30	−0.12
**Attention **	−0.09	0.13	−0.47*	−0.09	0.02	−0.38
**Working memory/flexibility**	−0.10	−0.28	−0.28	−0.29	−0.33	−0.07

CORT, cortisol; TEST, testosterone; PROG, progesterone; DHEA, dehydroepiandrostendione; DHT, dihydrotestosterone; DOC, 11-deoxycorticosterone. *Significant correlation.

## Discussion

Our results confirmed the presence of global cognitive deficit in first-episode schizophrenia patients (FES). This finding, impaired cognition across all studied domains, as compared with healthy controls, is in full agreement with published research (39, 40). Additionally, functional correlates of cognitive deficit in FES have been reported recently (41). Cognitive dysfunction, albeit less pronounced than in patients, has been previously described among healthy first-degree relatives as well (42). Cognitive functioning is therefore considered as one of the most consistent endophenotypes of psychotic disorders (43).

Notably, our results strongly suggest that the endophenotype is not the global cognitive functioning but more likely individual, specific domains of cognition. We found verbal memory and abstraction significantly impaired in both patients and siblings compared with controls. Moreover, the performance in verbal memory and abstraction did not differ between patients and siblings; thus, both domains can be viewed as candidate intermediate endophenotypes (26, 44). The results further corroborate findings of other authors who identified executive control functions (i.e., abstraction and flexibility) and verbal memory as the most impaired cognitive domains that did not differ between unaffected siblings and patients with psychotic or bipolar disorder (42, 45, 46).

Analysis of NAS revealed significantly higher plasma levels of cortisol, testosterone, and dihydrotestosterone in siblings as compared with healthy controls. Siblings also had higher levels of testosterone and dihydrotestosterone than had patients. Somewhat surprisingly, no difference in NAS between patients and controls was observed, except for the higher levels of DHT in controls. Since all FES were already treated with antipsychotics, an effect of medication on steroid levels cannot be completely ruled out. Previous studies demonstrated the impact of second-generation antipsychotics on NAS levels in rodent models (17, 47). Decreased testosterone in our FES group can hence be related to administration of antipsychotic drugs (48). While the small number of patients in our study and the heterogeneity of drugs used do not allow more detailed analysis of the impact of specific antipsychotics on the NAS levels, it should be acknowledged that the length of drug treatment was in fact very short (median 1.38 months).

Observed higher levels of testosterone and its active metabolite dihydrotestosterone among healthy siblings seem to be in contrast to previously reported data (49). Nonetheless, in some models, high testosterone has been associated with a hyperdopaminergic state, a presumptive high-risk state for development of psychosis (50, 51). A recent meta-analysis also confirmed higher testosterone levels in FES (15). Increased levels of cortisol in siblings are consistent with published literature, since higher cortisol levels have been repeatedly found among high-risk populations (52, 53) and are associated with higher transition rate into psychosis (54).

Cognition is a very intricate and complex neural process, based on synaptic plasticity, connectivity, and density. Various agents, including neurosteroids, can affect its neurodevelopment (55). It has been discussed that particularly DHEA and its sulfate conjugate play an important role in the development, neuroprotection, and restoration of neuronal characteristics through aging, although the studies with supplementation demonstrated only a small effect (56).

Despite the small sample size, we were able to identify several interactions between the cognitive performance and neurosteroids. The only cognitive domain not affected by steroid levels was attention. The most notable impact of NAS was on all domains of memory functions: Verbal memory was significantly influenced by CORT, TEST, DHEA, DHT, and DOC; visual memory by CORT, TEST, PROG, DHEA, DHT, and DOC; and working memory by CORT and DHT. Abstraction interacted with TEST, PROG, and DHT, and speed of processing interacted only with DHT. Similarly, other authors consistently reported positive effects of testosterone on memory function (including enhancement of hippocampal plasticity), and the effects of progesterone differ according to acute or chronic administration (55, 57).

While no significant correlation was observed in healthy individuals, cortisol levels in patients were associated with better performance in processing speed and working memory, which is in contrast with previous findings that associated higher CORT levels with poorer cognitive performance (34, 35, 58). Progesterone levels in patients were negatively associated with abstraction. In siblings, testosterone correlated negatively with verbal memory and progesterone with attention. The role of progesterone is rarely investigated; a single study identified a correlation between PROG and cognitive functioning in male schizophrenia patients (38).

Previous findings in healthy subjects underlined the higher impact of NAS on cognition in men than in women (59). For example, the role of testosterone in cognitive impairment has been discussed in male patients with schizophrenia only (37). Clearly, the effects of NAS levels on cognitive functioning should be interpreted according to gender. Due to the limited sample size, we were not able to analyze the gender difference in study groups.

In summary, we were able to endorse cognitive deficit as an endophenotype of psychotic disorder. While the results are still preliminary, the major finding is the observation that most likely not a global dysfunction but individual domains of cognition (i.e., abstraction and verbal memory) might be the foundation of cognitive endophenotype. Impairment in specific cognitive domains is shared by both first-episode patients and their healthy siblings and distinguishes them from the general population.

There are several study limitations (primarily small sample size, cross-sectional design without follow-up, and patients who were not drug naive) that prevent us from any generalization of neurosteroid data and do not allow for their plasma levels to be established unequivocally as an endophenotype of schizophrenia. We were not able to verify or reject steroid metabolome as a diagnostic tool, as suggested previously (60). Nevertheless, higher levels of cortisol and testosterone in siblings are consistent with high-risk states for psychosis, and cortisol levels are associated with higher transition rates to fully develop psychosis. Finally, our study results revealed in both patients and siblings multiple interactions between NAS and cognitive functioning, especially memory functions. If replicated in a larger study sample, this observation could have potential implications for timely therapeutic interventions in high-risk individuals, prodromal phases, and early phases of psychotic disorders.

## Ethics Statement

The study protocol was approved by the Ethical Committee of the National Institute of Mental Health, Czechia. All study subjects signed an informed consent form.

## Author Contributions

PK collected clinical and biochemical data, recruited subjects, and drafted the manuscript. VV collected and analyzed neuropsychological data and recruited subjects. AD and PS collected neuropsychological data. MR designed neuropsychological testing and interpreted cognitive data. AC performed statistical analyses. EK and MK analyzed biochemical samples. ZK designed neuropsychological testing. SC collected clinical data. PM drafted the study design, interpreted the results, and drafted the manuscript.

## Funding

The study was supported by grant from the Czech Ministry of Health, no. AZV 15-28998A; by grant from the Czech Science Foundation, no. 16-13093S; and by the project NPU4NUDZ LO1611 of the Czech Ministry of Education, Youth and Sports.

## Conflict of Interest Statement

The authors declare that the research was conducted in the absence of any commercial or financial relationships that could be construed as a potential conflict of interest.
